# Scleral exposure influences social judgments of trustworthiness, attractiveness, sociability, and social rank in White faces

**DOI:** 10.1371/journal.pone.0348193

**Published:** 2026-05-12

**Authors:** Mathias Boyer-Brosseau, Simon Rigoulot, Sébastien Hétu

**Affiliations:** 1 Department of Psychology, Université du Québec à Trois-Rivières, Trois-Rivières, Québec, Canada; 2 CogNAC Research Group, Université du Québec à Trois-Rivières, Trois-Rivières, Québec, Canada; 3 NECS laboratory, Université de Montréal, Montréal, Québec, Canada; 4 Department of Psychology, Université de Montréal, Montréal, Québec, Canada; Save Sight Institute, AUSTRALIA

## Abstract

Faces are powerful sources of social information, and the eye region plays a central role in shaping first impressions. While prior studies have suggested that scleral exposure may influence social judgments, findings remain inconsistent, and experimental approaches, nonexistent. The present study employed a controlled experimental design to explore how scleral exposure might influence judgments of trustworthiness, attractiveness, sociability, and perceived social rank in peer-related facial perception. A total of 162 participants rated 50 neutral faces twice, each digitally manipulated to display small and large scleral exposure. Results showed a consistent “scleral exposure effect”: across all measured social judgments, faces with larger scleral exposure were rated more favorably than the same faces with smaller scleral exposure. No interactions emerged between scleral exposure and sex of the raters or sex of the presented faces, indicating that these scleral exposure effects may reflect general sex-independent mechanisms. These findings provide the first experimental evidence that scleral exposure shapes multiple dimensions of social judgment, favoring greater exposure.

## Introduction

Human faces are a rich source of social information. Even in the absence of overt emotional expression, faces trigger automatic emotional responses that guide our everyday social interactions, taking the form of first impressions or social judgments [1]. Beyond the rapid processing of basic physical features such as age, ethnicity, and gender, when we look upon a face, we simultaneously evaluate impressions of trustworthiness, attractiveness, likeability, and competence [2–5]. These judgments emerge within milliseconds, often occurring outside of conscious awareness [2–5]. These processes likely serve adaptive functions, helping individuals to navigate their social environments, evaluating the outcomes of their interactions, avoiding potential threats, and facilitating approach toward socially beneficial peers [6,7].

The way facial traits shape social judgments is still poorly understood. Facial processing is both holistic and feature-dependent, as each specific region plays an individual role [8,9], yet traits seem to interact with each other to produce a single processed unit [10–12]. When looking at single facial regions, studies tend to show that in neutral faces specifically, the eye regions are the most important regarding judgments of trust and attractiveness [9,13]. But we still know little about how and why different features of the eye drive first social judgments.

Previous work has examined the role of several ocular traits in social perception, including limbal rings [14,15], iris coloration [16,17], pupil size [18], as well as interactions between these features [19,20]. Importantly, recent investigations have highlighted the relevance of the peri-iridial regions, *i.e.,* scleral regions (the “white” part of the eye), in social judgments. Globally, these studies suggest that increased scleral lightness is associated with more positive evaluations, including higher ratings of trustworthiness, attractiveness, and cooperativeness [21–24], although recent research seems to show adverse results when accounting for sex-typicality [25].

Scleral exposure (the amount of visible “white” in the eye) also seems to play a role in impression formations, but its role remains underexplored. A previous study exploring the effects of scleral exposure, sex, and race on perception of trust found significant associations, but only in White female raters, where average levels of scleral exposure were preferred [26]. These researchers later found no significant association between scleral exposure and common markers of attractiveness [27]. However, in these studies, scleral exposure was assessed through the Sclera Size Index (SSI), where the width of the exposed eyeball is divided by the diameter of the iris, and Boyer-Brosseau et al. [28] highlighted that this measurement might not be reliable enough to be used in these research contexts. When using different measurements, such as the Scleral Area Ratio (SAR), where the scleral areas are divided by the entire eye areas, significant associations between scleral exposure and judgments of trust and attractiveness were found, where averaged scleral exposure was globally associated with higher ratings of trust and attractiveness, highlighting the unclear role of scleral exposure and social perception.

Regardless, previous studies on scleral exposure and social perception were correlational and based on uncontrolled stimuli, limiting any causal interpretation. Potential confounding variables such as the sex of the presented face, scleral lightness, iris coloration, pupil dilation, or any other facial feature were not systematically controlled, which may have obscured effects of scleral exposure on social judgment.

The present study addresses most of these limitations by using an experimental approach, where apparent scleral size is systematically manipulated while other ocular and facial features are controlled. Specifically, we designed a facial judgment task in which participants evaluated faces on four dimensions: trustworthiness, attractiveness, sociability, and social rank. For each face, the eye region was replaced with one of two realistic eye models: one with small scleral exposure and one with large scleral exposure.

An important body of literature points to a preference towards salient and visible peri-iridial regions [21,24,28]. Several frameworks have been proposed to explain preferences related to human eye morphology. These include hypotheses regarding the role of scleral visibility in facilitating gaze following [24,29,30] and cooperative interactions [31,32], biases favoring saliency [8,33,34], and accounts linking specific traits to underlying biological quality [21], or neoteny [35]. These perspectives are not mutually exclusive, and current empirical evidence does not converge on a single explanatory account. Accordingly, the present study does not aim to adjudicate between these evolutionary explanations but rather to provide experimental evidence for an effect that felt underexplored.

Based on previous results, we hypothesized that in our experiment, faces with large scleral exposure would elicit higher ratings of each measured social judgment than faces with small scleral exposure and that this effect would be independent of the sex of participants [27] and of the presented face [28].

## Methods

### Participants

200 participants were recruited. The sampling was conditioned by two inclusion criteria: being at least 18 years old and being a citizen of either the United States or Canada. The reason we restricted recruitment to American and Canadian citizens was to limit the potential influence of cultural differences on social judgments. All participants were recruited via the MTurk (Amazon Mechanical Turk) platform from the first to the third of July 2022. Data was initially accessed on the third of July 2022. Participation was anonymous, and no information that could identify participants was available during or after data collection. A compensation of $2.00 USD was offered upon completion of the task. For more descriptive statistics of the sample, see the Results section.

### Stimuli

**Fig 1:**
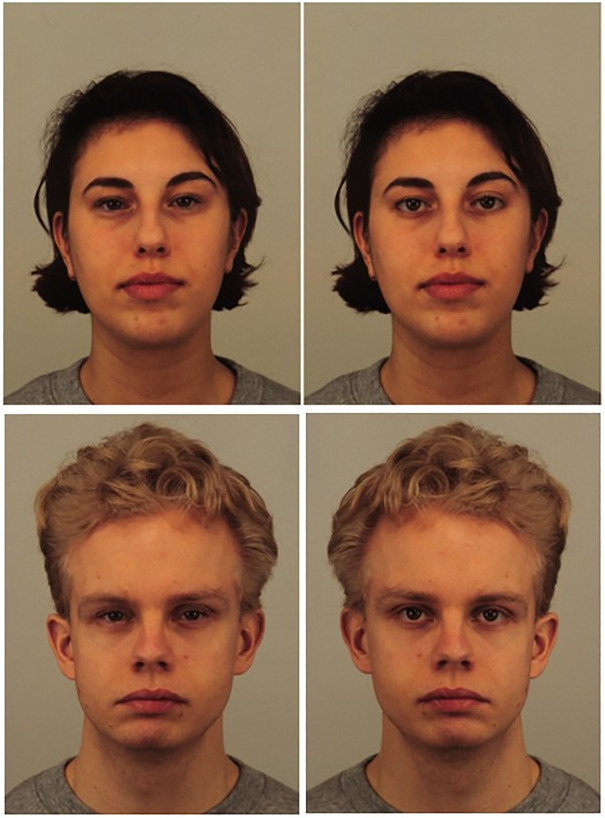
Example of a stimulus pair used in the experiment (AF17 and AM05) illustrating small (left) and large (right) scleral size. **Original, unmodified images are from the Karolinska Directed Emotional Faces database.** Reprinted from the Karolinska Directed Emotional Faces under a CC BY license, with permission from Karolinska Institutet, Psychology section, original copyright 2026.

The facial stimuli used in the experimental task came from the Karolinska Directed Emotional Faces (KDEF) database [36]. For this study, 25 male and 25 female faces with neutral expressions were randomly selected (alongside 2 additional faces for the practice session and 3 others for manipulation checks). For each face, the eye regions were manually replaced twice, with two distinct pairs of eyes: one with a “small” scleral exposure and one with a “large” scleral exposure (see Fig 1). Manipulated eye models came from unused faces of the same database. Scleral exposure was chosen to appear natural, assessed using SAR and is expressed as the percentage of scleral area per total eye area, averaged for both eyes. The selection of eye models was primarily guided by the principle that the eye region should be clearly visible (e.g., minimal makeup, no eyelashes obstructing scleral visibility). We aimed to represent naturally occurring conditions of small and large scleral exposure. To achieve this, we measured the SAR in the white subsets of both the Chicago Faces Database (CFD) [37] and KDEF facial databases [36]. We then selected SAR values closest to the first (35.96%) and third quartiles (44.91%) of the distribution, ensuring that the chosen models represented valid small and large exposure conditions while avoiding outliers or unusually extreme values (see supporting information). Thus, SAR in the small scleral condition is 31.5%, and SAR for the large scleral condition is 43.8%. Trial presentation was pseudorandomized for each participant, ensuring a random order while preventing the same face from appearing in immediate succession across different scleral sizes. The selected eye models were matched for comparable elongation and ensured to be clearly visible, free from obstructions such as makeup or eyelashes. A single model of iris and pupil was used across all eye models, and the average luminance values of scleral areas were corrected to ensure homogeneity between conditions. All digital modifications were manually performed using Adobe Photoshop [38], and eye measurements were manually performed using the measurement tool from the open-source software ImageJ [39].

### Task

The main task was preceded by an information and consent form, main instructions, and a clear definition of social ranking, *i.e.,* the MacArthur Scale of Subjective Social Status – Adult Version [40] (see supporting information). It was then followed by a practice round, containing two trials and an attentional catch trial that were not included in the main analyses.

The experimental task was conducted online via the LimeSurvey platform. Participants had to rate individuals based on photos of their faces. In total, 100 visual stimuli were presented, all displaying a neutral facial expression. These stimuli were divided into two conditions: faces with small scleral exposure (n = 50) and faces with large scleral exposure (n = 50). On each trial, a face stimulus (200 x 271 pixels) appeared and remained at the center of the participant’s screen, along with an interactive response box located directly below the image. This box allowed participants to report their social judgments. Four questions were presented in the response box:

1. “How much would you trust this person?”
2. “Would you interact with this person?”
3. “Do you find this person physically attractive?
4. “According to you, what would be the social rank of this person within our society?

These questions respectively aimed to assess perceived trustworthiness, sociability, physical attractiveness, and perceived social rank. Participants responded using a 10-point Likert scale. Each trial’s duration was self-paced, with participants clicking a “Next” interactive button at the bottom of the page to proceed to the following stimulus. To identify automated or inattentive responses, two attention check trials were embedded within the experimental procedure. One attention check was randomly placed within the first block of 50 faces, and another within the second block. These checks followed the same format as the experimental trials: a face not used in the main task was shown at the center of the screen with an interactive response box below. Participants were asked to specify whether the face presented was male or female. The first attention check featured a male face, and the second a female face. Participants who failed either of the two attention check trials were excluded from the analysis.

Following the task, a mandatory sociodemographic questionnaire was presented for descriptive purposes (see supporting information).

### Ethics statement

This study was approved by the CERPPE Ethics Committee of the Université du Québec à Trois-Rivières (certificate CERPPE-22-09-07.14). Participants provided written informed consent prior to participation by selecting a checkbox before beginning the task.

### Analysis

To identify potential outliers at the participant level, we computed the mean rating across all trials for each dependent variable (trustworthiness, sociability, attractiveness, and social rank). Outlier detection was performed using the Median Absolute Deviation (MAD) method (k = 2.5), for its robustness over methods using standard deviation around the mean [41]. This procedure was applied independently for each dependent variable, and participants flagged as outliers in any of the variables were excluded from the analyses.

Linear mixed models were used to assess the influence of scleral exposure on participants’ ratings. This parametric approach was selected following Huh & Gim’s guidelines suggesting that ordinal data with at least five approximately equal intervals can be analyzed as continuous without a meaningful increase in Type I error [42]. Separate models were fit for each dependent variable—trustworthiness, attractiveness, sociability, and social rank—with fixed effects for scleral exposure (large as baseline), sex of the rated face (female as baseline), and sex of the participant (male as baseline). Our models allowed random intercepts for participants and stimuli ID, as well as a random intercept accounting for scleral exposure at the stimulus level. For each social rating, models with main effects only and models including two-way or three-way interactions were compared. Model selection was made with a goodness-of-fit approach, using both the Akaike Information Criterion (AIC) and the Bayesian Information Criterion (BIC), as they penalize model complexity differently [43]. For each social rating, models with main effects only and models including two-way or three-way interactions were compared. Differences in AIC were interpreted following Burnham and Anderson’s guidelines [44], where ΔAIC values of approximately 0–2 indicate weak evidence, 4–7 moderate evidence, and values greater than 10 indicate strong evidence in favor of the model with the lower AIC. Differences in BIC were interpreted following Kass and Raftery [45], whereby ΔBIC values of 0–2 indicate weak evidence, 2–6 positive evidence, 6–10 strong evidence, and values greater than 10 very strong evidence in favor of the model with the lower BIC. When AIC and BIC converged on the same model, that model was retained. When they diverged, the more complex model was retained only when gains in AIC were substantial and BIC penalties remained moderate (and vice versa). All analyses were made with R [46] using RStudio [47]. Models were estimated using the *lmer* function from the *lmerTest* package [48]. Effect sizes were calculated as Cohen’s *d*, using the *sjstats* package [49]. Estimated marginal means and pairwise contrasts were computed using the *emmeans* package [50]. Plotting was done using the *ggplot2* package [51].

## Results

Out of the initial 200 participants, 36 were excluded for failure at one of the 2 attentional check trials. An additional 2 were later removed from MAD outlier detection, for a total of 162 participants. Out of these subjects, each one reported being from the United States; 107 reported being men (66.1%) and 55 women (33.9%). Age distribution was on average 39.3 years old (SD = 11.3). 145 reported being of White ethnicity (89.5%), 9 African (5.6%), 2 Latino (1.2%), and 6 Other/Prefer not to answer (3.6%).

### Model selection

Goodness of fit analysis indicated that the inclusion of interaction terms between the sex of the participant and the sex of the rated face improved model fit for trustworthiness, attractiveness, sociability, and social rank. The full dataset used is available in the supporting information (S1 Table).

### Social ratings

**Fig 2:**
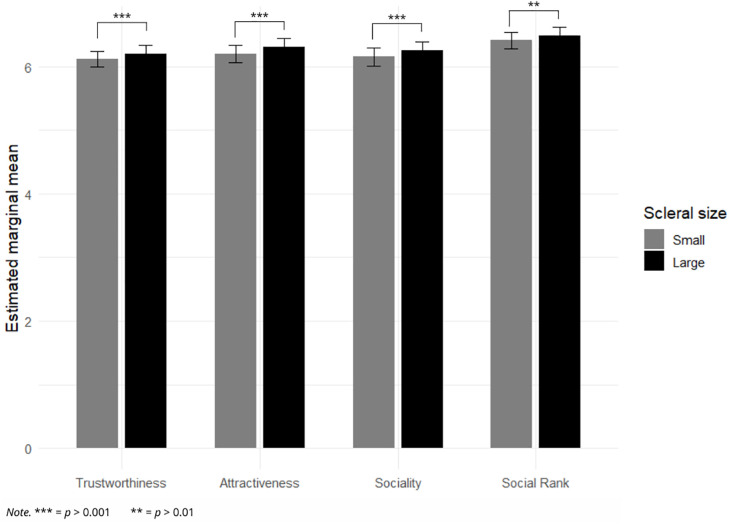
Estimated marginal means of social judgment scores by scleral exposure.

Across all measured social ratings (trustworthiness, attractiveness, sociability, and social rank), scleral exposure had a significant effect. Faces with large scleral exposure were rated more positively than faces with small scleral exposure (βs < 0, *p*s ≤ 0.007). A consistent interaction between the sex of the rated face and the sex of the participant was also observed across all outcomes (*p*s ≤ 0.001). Follow-up estimated marginal means indicated that male faces were judged less positively than female faces by both male and female participants, with this difference being systematically stronger among female participants. Results of scleral exposure effects on each rated social judgment are detailed below and presented in Fig 2. Detailed contrasts and interaction plots are provided in the online supplementary materials (OSF repository).

### Trustworthiness

Results indicated a significant main effect of scleral exposure on perceived trustworthiness (β = −0.084, SE = 0.023, *t*(50) = −3.66, *p* < 0.001). Estimated marginal means indicated higher trustworthiness ratings for faces with large scleral exposure (M = 6.20, SE = 0.13) compared to faces with small scleral exposure (M = 6.12, SE = 0.13), *z* = 3.66, *p* < 0.001. A significant interaction was observed between the sex of the rated face and the sex of the participant (β = −0.160, SE = 0.038, *t*(15,940) = −4.21, *p* < 0.001). Follow-up estima*t*ed marginal means showed that male faces were judged as less trustworthy than female faces by both male participants (*z* = 3.18, *p* = 0.002) and female participants (*z* = 5.62, *p* < 0.001), with this difference being more pronounced among female participants.

### Attractiveness

Results indicated a significant main effect of scleral exposure on perceived attractiveness (β = −0.107, SE = 0.026, *t*(50) = −4.09, *p* < 0.001). Estimated marginal means indicated higher attractiveness ratings for faces with large scleral exposure (M = 6.31, SE = 0.13) compared to faces with small scleral exposure (M = 6.20, SE = 0.13), *z* = 4.09, *p* < 0.001. A significant interaction was observed between the sex of the rated face and the sex of the participant (β = −0.188, SE = 0.042, *t*(15,940) = −4.47, *p* < 0.001). Follow-up estima*t*ed marginal means showed that male faces were judged as less attractive than female faces by both male participants (*z* = 4.13, *p* < 0.001) and female participants (*z* = 6.62, *p* < 0.001), with this difference being more pronounced among female participants.

### Sociability

Results indicated a significant main effect of scleral exposure on perceived sociability (β = −0.094, SE = 0.026, *t*(50) = −3.57, *p* < 0.001). Estimated marginal means indicated higher sociability ratings for faces with large scleral exposure (M = 6.25, SE = 0.14) compared to faces with small scleral exposure (M = 6.16, SE = 0.14), *z* = 3.57, *p* < 0.001. A significant interaction was observed between the sex of the rated face and the sex of the participant (β = −0.226, SE = 0.041, *t*(15,940) = −5.57, *p* < 0.001). Follow-up estima*t*ed marginal means showed that male faces were judged as less social than female faces by both male participants (*z* = 2.70, *p* = 0.007) and female participants (*z* = 6.14, *p* < 0.001), with this difference being more pronounced among female participants.

### Social rank

Results indicated a significant main effect of scleral exposure on perceived social rank (β = −0.077, SE = 0.028, *t*(50) = −2.80, *p* = 0.007). Estimated marginal means indicated higher social rank ratings for faces with large scleral exposure compared to faces with small scleral exposure. A significant interaction was observed between the sex of the rated face and the sex of the participant (β = −0.129, SE = 0.039, *t*(15,940) = −3.30, *p* = 0.001). Follow-up estimated marginal means showed that male faces were judged as having lower social rank than female faces by both male participants (*z* = 2.29, *p* = 0.022) and female participants (*z* = 4.21, *p* < 0.001), with this difference being more pronounced among female participants.

## Discussion

The objective of this study was to explore the influence of scleral exposure on social judgments. To the best of our knowledge, it is the first to do so in an experimentally controlled design, building upon previous similar works [26–28,52]. We hypothesized that faces with larger exposed scleral areas would elicit higher ratings of trustworthiness, attractiveness, sociability, and social rank, without interacting with the rater’s sex or the sex of the stimuli. Accordingly, our results indicated that on average, faces with larger scleral exposure globally elicited higher ratings than identical faces with small scleral exposure. This “scleral exposure effect” was not modulated by either the sex of the participant or the sex of the rated face. However, we found that female faces were rated more positively than male faces, with this difference being more pronounced among female participants.

Our findings provide novel evidence for a global “scleral exposure effect.” While prior research suggested that scleral exposure influences judgments of trustworthiness and attractiveness [26,28], our results show that this preference extends to perceptions of sociability and social rank. Our results show a significant advantage of larger over smaller scleral exposure on those judgments. However, our protocol did not allow us to directly test the nonlinearity of those effects, as previous work on scleral exposure highlighted [26,28], nor whether averaged scleral exposure would be optimally preferred, as facial averageness theory would predict [53]. This remains an interesting future avenue.

The absence of an interaction between scleral exposure and sex of the rated face or sex of the participant is intriguing because, in white populations at least, scleral exposure is sexually dimorphic; men exhibit larger exposed sclerae than women do [52]. Such dimorphisms have been hypothesized to function as ornamented cues in mate choice, exaggerated sexual traits, that might be used to attract mates [52]. Yet, our results show that large scleral exposure is generally preferred across both sexes, without any sex-specific effects. This absence of sex-related effects was previously highlighted regarding attractiveness judgments [27], but our results extend that claim by suggesting that scleral exposure might act more as a universal social cue. The generalized preference for larger sclerae is also consistent with alternative hypotheses regarding scleral lightness, suggesting that highly visible scleral regions are socially favored [23,24] and that this might be because it allows for easier gaze identification [30,54,55]. Indeed, humans are highly sensitive to others’ eyes. We are naturally drawn to them, and even from infancy, we seek to follow another’s gaze [31,56]. Gaze following appears to be a crucial component of our social environment: it allows us to infer where others’ attention is directed [57–59], to interpret implicit attitudes and expectations during social interactions [60–63], and to anticipate others’ behaviors [64]. It has been shown that larger scleral openings also enhance the accuracy of gaze following [65], and our findings may reflect a similar underlying process. Although we have not tested for this directly, our results may fall in line with Wacewicz et al.’s [24] proposal that individuals with lighter sclerae may be perceived as more desirable social partners, as their gaze is easier to track, and that their intents or their behaviors might be easier to predict. Our study may expand the scope of this proposal to include scleral exposure to the factors that might make it more visible. This proposal is often associated, in the current literature, with communicative-based evolutionary frameworks involving sexual selection, such as the cooperative eye hypothesis [29,31]. However, the evolutionary processes shaping ocular morphology in humans remain highly debated, especially regarding communicative function [66,67]. In primates, new evidence points toward photoprotective and diverse ecological factors instead [68,69]. Another possible explanation for these results may relate to possible neotenic processes. Indeed, ocular morphology varies across the lifespan, with age-related changes impacting the palpebral fissure and the overall visibility of the exposed eye region [70]. While infants and children do not exhibit maximal scleral exposure per se [71], scleral exposure typically peaks around the ages of 20–25 [72]. It is followed by a progressive reduction in later adulthood, mainly due to a softening of the upper periorbital tissues [73]. As such, increased scleral exposure may be perceived as a visual cue to youthfulness and health. A similar age-related pattern has also been highlighted for peri-iridial coloration as well [21,24,74].

No additional interaction between scleral exposure and sex of the participant was highlighted for trust and attractiveness ratings. This aligns with our previous findings [28], but contrasts with Danel et al. [26], who previously reported an interaction between the sex of the participants and scleral size on ratings of trust. The most probable explanations for this might lie in methodological differences. In that study, scleral exposure was assessed using the Sclera Size Index (SSI), a metric that has been widely used in the literature. However, recent work has suggested that this measure may have limitations due to its unidimensional nature and that area-based indices, such as the Scleral Area Ratio, may provide a more reliable representation of scleral exposure [28]. Moreover, to the best of our understanding, both of these studies employed linear modelling with similar repeated-measures correlational designs, though the first study [26] did not account for random effects. In such cases, observations were probably nested within participants and stimuli, and ignoring this structure could have resulted in an increase in Type 1 error chance. Nonetheless, given the small sample of studies on the matter, conclusions should be considered provisional.

### Limitations

Several limitations of our study should be noted. Firstly, as Fiala et al. [25] noted, social perception, especially regarding ocular morphology, has predominantly been researched within White, Western populations. Unfortunately, our study aligns with this body of literature. While we aimed at minimizing cultural biases by presenting faces of White individuals and restricting our sample to participants from the United States. The risk of isolating ethno-specific effects was amplified by the final demographic of our sampling, which was composed of a White majority. The results of our study thus inevitably highlight population-specific effects. Whether these “scleral exposure effects” generalize to other populations remains an important question for future research.

Secondly, we did not control for total eye size, thus the total eye area of the large scleral exposure model was naturally bigger than the small scleral exposure model. This constraint could impact our results, particularly as changes in eye size influence social judgment altogether [75,76]. Our objective in this study was to maintain ecological validity and to present “natural”, real-like eye models. Specifically, the eye models used were real, unmanipulated eyes from individuals whose scleral exposure levels fell within a non-extreme distribution. Digital manipulations consisted solely of transplanting these eyes onto facial stimuli. In this scenario, controlling for total eye size would mean manipulating the iris area as it is often done in the associated literature [8], and we feared these eye models would feel unnatural or unrealistic. If multicollinearity issues regarding total eye size measurements and scleral exposure measurements are taken into account, we believe this constraint might be avoided for future studies.

Thirdly, although all faces and eye models used were validated as having neutral expressions, altering the eye models may have unintentionally created stimuli conveying emotional displays. For example, faces with smaller scleral exposure might have been perceived as having narrowed eyes, which could be interpreted as expressing emotions such as disgust or anger [77,78]. We did not specifically control for or measure this potential confound. In a similar sense, while we attempted to maintain consistent eye elongation across stimuli, we could not fully control for variations in eye shape. Although we aimed at selecting eye models that visibly presented similar shapes, differences such as rounder versus narrower eyes may influence perception and interact with scleral exposure in ways that were not directly accounted for in our design. An effective way to address this could be by reassessing perceived emotional display post stimuli modification. Another way could be by employing a geometric morphometrics-based design. This approach could quantify eye shape geometry using landmarks, allowing us to isolate the interaction of eye shape on judgments. This was previously explored by Kleisner et al. in their study on iris coloration [17] and Lewandowski et al. [79] on human-like stimuli.

Research on social perception (and gaze cueing) has often focused on single ocular features in isolation, such as scleral brightness, pupil size, iris brightness, or limbal rings. In everyday perception, however, these features are likely to influence one another, on social perception (and on eye saliency). Our results show that scleral exposure on its own is sufficient to affect social evaluations. At the same time, recent findings indicate that some ocular cues interact in more complex, non-additive ways, as shown for pupil size and iris brightness [80,81]. In this sense, Perea-García et al. [20] and Howard [80] argue that traits shouldn’t be assessed in isolation. Although the present study did not look at such interactions, isolating scleral exposure while keeping other eye features constant allowed us to show that exposure itself plays a meaningful role in shaping social perception. While it remains informative to isolate single trait effects, studying how each trait may interact with one another to influence social perception remains a crucial next step.

Finally, supplementary analyses revealed a high positive correlation between ratings of trustworthiness, attractiveness, sociability, and social rank (*r*s ranged from 0.67 to 0.79). Although conceptually distinct, these judgments are not independent and tend to co-vary in the context of first impressions. In this sense, individuals that are perceived as more attractive tend to be attributed more favorable traits, such as trust and sociability, as well as intelligence, competence, and emotional stability [82]. This is often referred to as the “attractiveness halo effect” [83]. These results are available in the Supporting Information (see S2 Table). Additional aggregated correlations of social ratings by presented face and by scleral exposure are available in the OSF repository.

## Conclusion

The present study experimentally examined the role of scleral exposure in shaping social judgments. Using a controlled online rating task, 162 participants evaluated 100 neutral faces that had been digitally manipulated to display either small or large scleral exposure. For each face, participants rated trustworthiness, attractiveness, sociability, and perceived social rank on a 10-point scale.

Our results provide novel evidence for a consistent “scleral exposure effect”: faces with larger scleral exposure were rated more positively across all social dimensions compared to identical faces with smaller scleral exposure. These effects did not vary according to the sex of the presented face or the sex of the participant, suggesting that scleral exposure exerts a general influence on social perception.

These findings highlight the potential role of scleral exposure as a cue for shaping social judgments. Yet, the modest effect sizes and absence of strong interactions indicate that scleral exposure probably operates in conjunction with other facial and contextual features, limiting conclusions about its significance in sexual evolution.

## Supporting information

S1 Fig SAR distribution with eye models. (PNG)

S2 Fig Task instructions and definitions. (JPG)

S1 Appendix Demographic questionnaire. (PDF)

S1 Table Model selection table. (DOCX)

S2 Table Correlation table. (DOCX)
