# Supplementary figures and images for: Scleral exposure influences social judgments of trustworthiness, attractiveness, sociability, and social rank in White faces
Source: PLoS One. 2026 May 12;21(5):e0348193. doi: 10.1371/journal.pone.0348193 (PMC13166915; doi:10.1371/journal.pone.0348193)

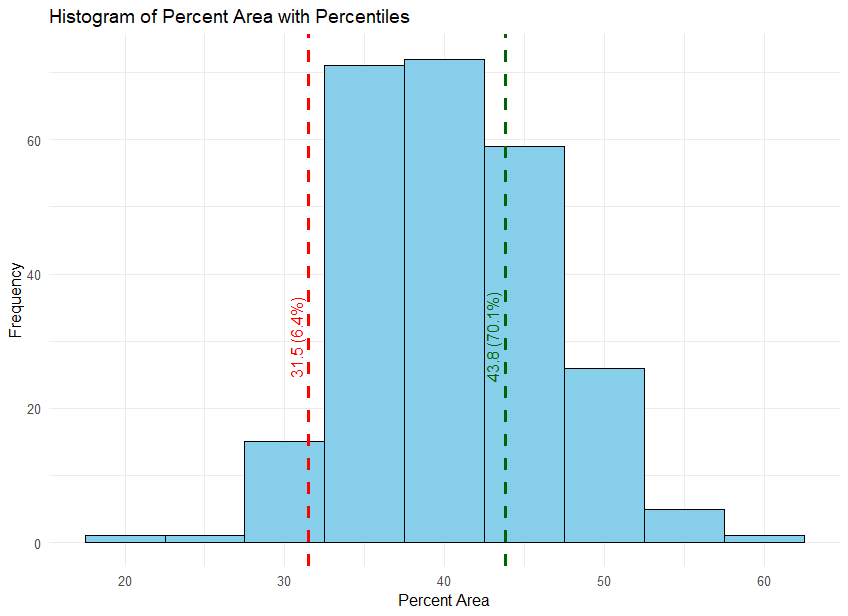

Supplement: S1 Fig — (PNG) [file pone.0348193.s001.png]

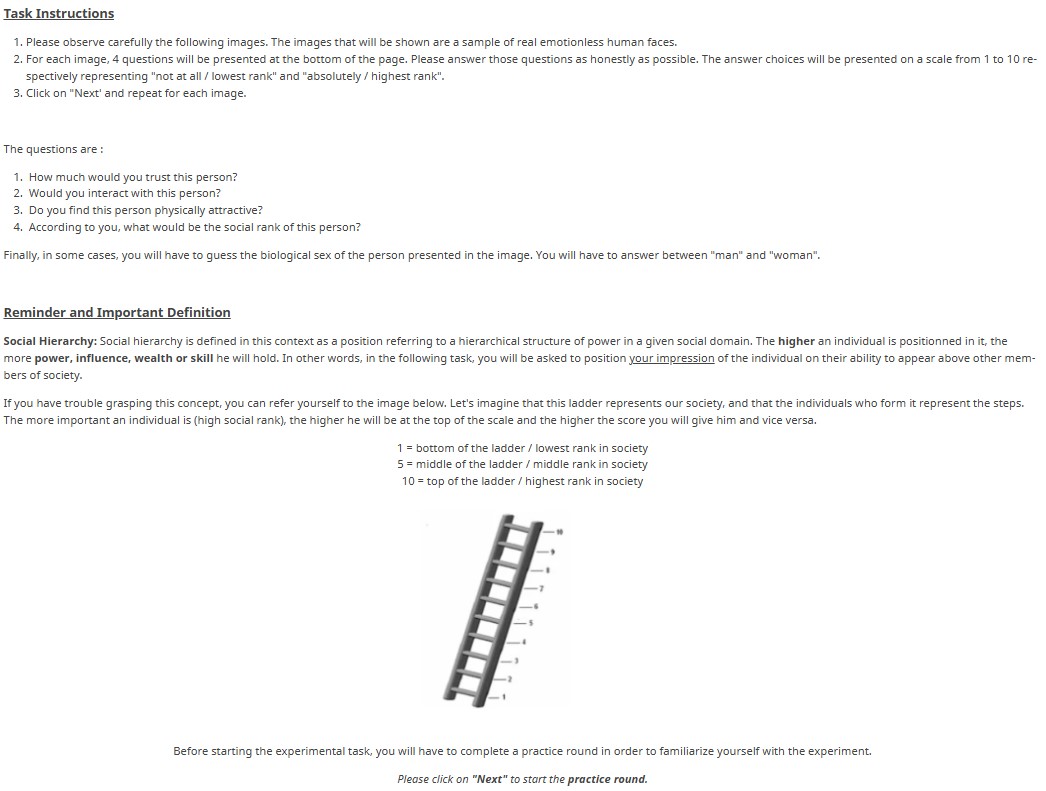

Supplement: S2 Fig — (JPG) [file pone.0348193.s002.jpg]
