# Supplementary material for: Scleral exposure influences social judgments of trustworthiness, attractiveness, sociability, and social rank in White faces
Source: PLoS One. 2026 May 12;21(5):e0348193. doi: 10.1371/journal.pone.0348193 (PMC13166915; doi:10.1371/journal.pone.0348193)
Supplement: S1 Appendix — (PDF) [file pone.0348193.s003.pdf]

Socio-Demographic Questionnaire

Please click on "Next" in order to start the mandatory socio-demographique questionnaire

* What is your age (in years) ?

Only numbers may be entered in this field.

* What is your cultural background?

Choose one of the following answers
If you choose 'Other:' please also specify your choice in the accompanying text field.

- European / White
- African
- Asian
- Hispanic / Latinx
- First nations / Indigenous
- Middle Eastern
- Prefer not to answer
- Other:

* What is your assigned sex at birth?

Choose one of the following answers
If you choose 'Other:' please also specify your choice in the accompanying text field.

- ○ Men
- ○ Woman
- ○ Prefer not to answer
- ○ Other: ____________

* What is your nationality?

Choose one of the following answers

Please choose...

* What is you civil status?

Choose one of the following answers

- ○ Married
- ○ Common law spouse
- ○ Seperated
- ○ Divorced
- ○ Widower
- ○ Single

- Prefer not to answer

*

What is your highest education level? (achieved or in progress)

Choose one of the following answers
If you choose 'Other:' please also specify your choice in the accompanying text field.

- Elementary
- High School
- Technical School
- Undergraduate Studies
- Master's Studies
- Doctorate's Studies
- Postdoctoral Studies
- Chose not to answer
- Other:

*

What is your main occupation?

Select all that apply

- Fullt-time work
- Part-time work
- Full-time student

- [ ] Unemployed
- [ ] Retired
- [ ] Authorized Leave
- [ ] Other:

*

What is on average your household annual income?

Choose one of the following answers

- Lower than 9 999 $
- 10 000 - 24 999 $
- 25 000 - 49 999 $
- 50 000 - 74 999 $
- 75 000 - 99 999 $
- 100 000 - 149 999 $
- 150 000 $ and higher
- Prefer not to answer

*

Consider that the ladder that we are showing represents the place that people occupy in society.

At the top of this ladder are the people who have more money, more education and better jobs.

At the bottom of the ladder are the people who have less money, less education and worse jobs (jobs with less recognition) or are unemployed.

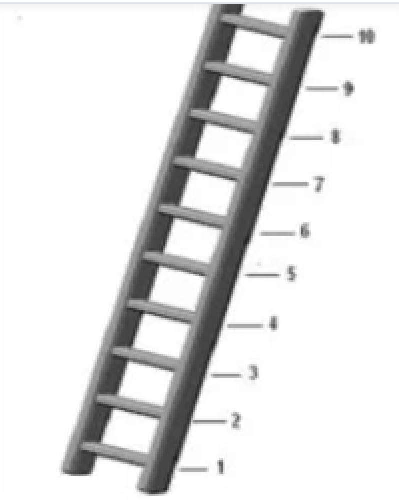

The higher you consider yourself in this ladder, the closer you will be to the people who are at the top of the ladder, and the lower, closer you will be to the people who find themselves at the bottom.

Where would you place yourself on this ladder?

Choose one of the following answers

Please choose...

*

Choose one of the following answers

- Smartphone
- Touchscreen tablet
- Computer
- Other:
