# Supplementary material for: Scleral exposure influences social judgments of trustworthiness, attractiveness, sociability, and social rank in White faces
Source: PLoS One. 2026 May 12;21(5):e0348193. doi: 10.1371/journal.pone.0348193 (PMC13166915; doi:10.1371/journal.pone.0348193)
Supplement: S1 Table — (DOCX) [file pone.0348193.s004.docx]

| dv | model | npar | AIC | BIC | logLik | dAIC | dBIC | AIC_support | BIC_evidence |
| --- | --- | --- | --- | --- | --- | --- | --- | --- | --- |
| Attirance | M3 | 9 | 54,614.00 | 54,683.23 | -27,298.00 | 0.00 | 0.00 | essentially equivalent (0–2) | weak (0–2) |
| Attirance | M0 | 8 | 54,631.92 | 54,693.46 | -27,307.96 | 17.92 | 10.23 | very weak support (>=10) | very strong (>=10) |
| Attirance | M2 | 9 | 54,631.90 | 54,701.13 | -27,306.95 | 17.90 | 17.90 | very weak support (>=10) | very strong (>=10) |
| Attirance | M1 | 9 | 54,633.91 | 54,703.15 | -27,307.96 | 19.91 | 19.91 | very weak support (>=10) | very strong (>=10) |
| Attirance | M4 | 12 | 54,617.96 | 54,710.28 | -27,296.98 | 3.97 | 27.04 | slightly worse (2–4) | very strong (>=10) |
| Confiance | M3 | 9 | 51,315.87 | 51,385.11 | -25,648.94 | 0.00 | 0.00 | essentially equivalent (0–2) | weak (0–2) |
| Confiance | M0 | 8 | 51,331.56 | 51,393.10 | -25,657.78 | 15.69 | 7.99 | very weak support (>=10) | strong (6–10) |
| Confiance | M2 | 9 | 51,332.27 | 51,401.51 | -25,657.14 | 16.40 | 16.40 | very weak support (>=10) | very strong (>=10) |
| Confiance | M1 | 9 | 51,333.55 | 51,402.79 | -25,657.78 | 17.68 | 17.68 | very weak support (>=10) | very strong (>=10) |
| Confiance | M4 | 12 | 51,320.52 | 51,412.83 | -25,648.26 | 4.65 | 27.73 | noticeably worse (4–7) | very strong (>=10) |
| Rank | M3 | 9 | 52,261.79 | 52,331.03 | -26,121.90 | 0.00 | 0.00 | essentially equivalent (0–2) | weak (0–2) |
| Rank | M0 | 8 | 52,270.68 | 52,332.22 | -26,127.34 | 8.88 | 1.19 | much worse (7–10) | weak (0–2) |
| Rank | M2 | 9 | 52,268.06 | 52,337.30 | -26,125.03 | 6.27 | 6.27 | noticeably worse (4–7) | strong (6–10) |
| Rank | M1 | 9 | 52,271.59 | 52,340.82 | -26,126.79 | 9.79 | 9.79 | much worse (7–10) | strong (6–10) |
| Rank | M4 | 12 | 52,262.04 | 52,354.35 | -26,119.02 | 0.25 | 23.33 | essentially equivalent (0–2) | very strong (>=10) |
| Socialite | M3 | 9 | 53,447.54 | 53,516.77 | -26,714.77 | 0.00 | 0.00 | essentially equivalent (0–2) | weak (0–2) |
| Socialite | M0 | 8 | 53,476.53 | 53,538.07 | -26,730.27 | 29.00 | 21.30 | very weak support (>=10) | very strong (>=10) |
| Socialite | M4 | 12 | 53,450.44 | 53,542.75 | -26,713.22 | 2.90 | 25.98 | slightly worse (2–4) | very strong (>=10) |
| Socialite | M2 | 9 | 53,475.87 | 53,545.10 | -26,728.93 | 28.33 | 28.33 | very weak support (>=10) | very strong (>=10) |
| Socialite | M1 | 9 | 53,478.34 | 53,547.58 | -26,730.17 | 30.81 | 30.81 | very weak support (>=10) | very strong (>=10) |

# Model Selection Results

Model comparison (top 5 models per dependent variable)

Note. Models are ordered by BIC within each dependent variable. AIC and BIC differences are reported relative to the best-fitting model.
