# Supplementary material for: Scleral exposure influences social judgments of trustworthiness, attractiveness, sociability, and social rank in White faces
Source: PLoS One. 2026 May 12;21(5):e0348193. doi: 10.1371/journal.pone.0348193 (PMC13166915; doi:10.1371/journal.pone.0348193)
Supplement: S2 Table — (DOCX) [file pone.0348193.s005.docx]

S1 Table. Correlation matrix of social judgment variables.

| Variable | Confiance | Attirance | Socialite | Rank |
| --- | --- | --- | --- | --- |
| Confiance | 1.000 | 0.734 | 0.768 | 0.685 |
| Attirance | 0.734 | 1.000 | 0.702 | 0.797 |
| Socialite | 0.768 | 0.702 | 1.000 | 0.666 |
| Rank | 0.685 | 0.797 | 0.666 | 1.000 |
